# Common Mistakes and Pitfalls in Magnetic Resonance Imaging of the Knee

**DOI:** 10.5334/jbr-btr.1206

**Published:** 2016-11-19

**Authors:** Filip Vanhoenacker, Nicolas De Vos, Pieter Van Dyck

**Affiliations:** 1 AZ Sint-Maarten and University (Hospital) Antwerp/Ghent, BE; 2 UZ Gent, BE; 3 UZ Antwerpen, BE

**Keywords:** Knee, MRI, Pitfalls, Variants, Interpretation errors

## Abstract

This pictorial review presents an overview of common interpretation errors and pitfalls in magnetic resonance imaging (MRI) of the knee. Instead of being exhaustive, we will emphasize those pitfalls that are most commonly encountered by young residents or less experienced radiologists.

## Introduction

Magnetic resonance imaging (MRI) of the knee joint is one of the most commonly requested in general radiological practise examinations and belongs to the core clinical practice in most MRI units along with spinal and brain MRI. Therefore, these examinations are often reported by general radiologists in most institutions and are an important part of routine education of radiology residents in clinical MRI.

Although interpretation of knee MRI seems straightforward in most scenarios, there are a number of pitfalls that may cause common mistakes. The purpose of this pictorial review is to present an overview of those common interpretation errors and pitfalls in MRI of the knee. We will particularly emphasize those pitfalls that are encountered by young residents or less experienced radiologists.

## Insufficient Knowledge of Developmental Anatomy and Ossification Variants

### Bipartite/multipartite patella

**Figure 1:**
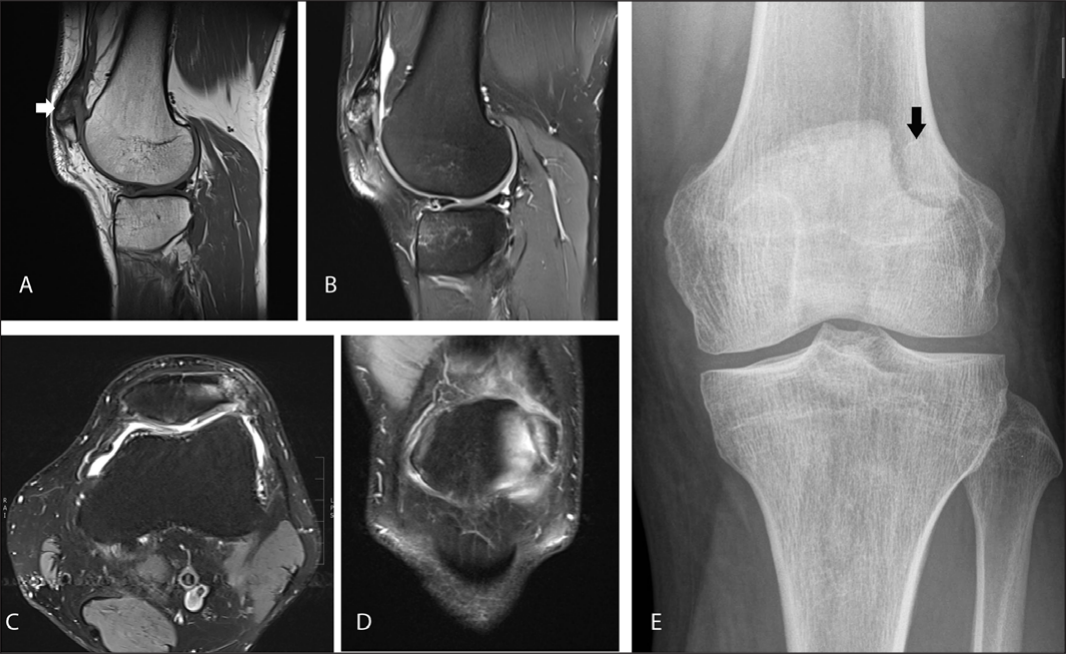
Symptomatic bipartite patella. Sagittal T1-WI **(A)** shows a separate bone fragment at the superolateral aspect of the patella (arrow). Sagittal **(B)**, axial **(C)** and coronal **(D)** FS T2-WI reveal bone marrow edema within the accessory fragment and the adjacent superolateral aspect of the patella. Plain radiography **(E)** confirms a sclerotic delineated accessory fragment at the superolateral aspect of the patella (arrow).

Bipartite or multipartite patella is the presence of one or more accessory ossification centers near the patella. In a previous study at our institution, we found a prevalence of 0.53% [1]. It may present as an incidental finding on imaging, but may be symptomatic and cause anterior knee pain. In symptomatic cases, there is often bone marrow edema at the accessory ossification center and the adjacent patella (Figure 1). It should not be confused with a patellar fracture [1]. Correlation with clinical history (acute onset of pain in fracture versus chronic knee pain in symptomatic bipartite or multipartite patella) is useful in the differential diagnosis. Plain films show a sclerotic delineation of the accessory ossification fragments in case of a bipartite or multipartite patella, whereas the borders are sharp in case of a fracture.

### Dorsal defect of the patella

**Figure 2:**
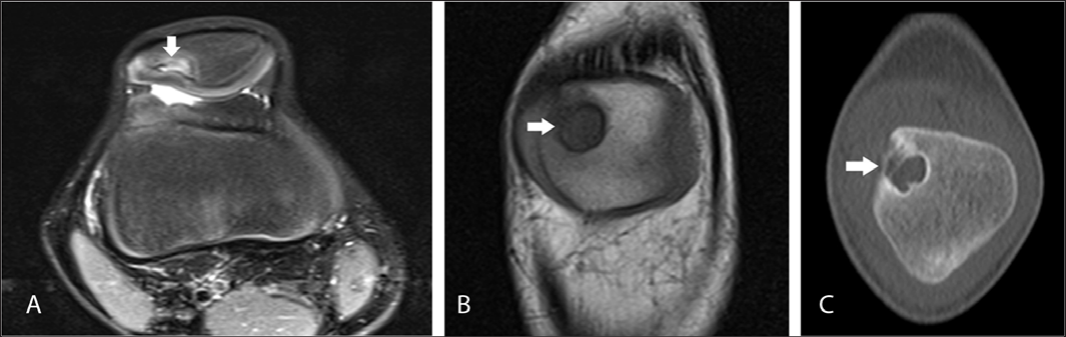
Dorsal defect of the patella. Axial FS T2-WI **(A)** and coronal T1-WI **(B)** show a well-delineated defect at the superolateral aspect of the right knee (arrow). The overlying articular cartilage is intact. On cone-beam CT **(C)**, the defect is sharply delineated and has a sclerotic border (arrow).

Dorsal defect of the patella (DDP) is a well-delineated defect at the articular side of the superolateral aspect of the patella (Figure 2). Retrospectively, this variation was seen in 0.16% of our MRI studies of the knee and may be bilateral [1]. On plain radiographs, DDP presents as a well-defined radiolucent lesion. It should not be confused with osteochondritis dissecans (OCD) of the patella, Brodie abscess or bone tumors. The clues to the correct diagnosis are the typical superolateral location and the intact smooth overlying cartilage. Most patients are asymptomatic, except in cases of overlying cartilage defects [2].

### Cortical avulsive irregularity

**Figure 3:**
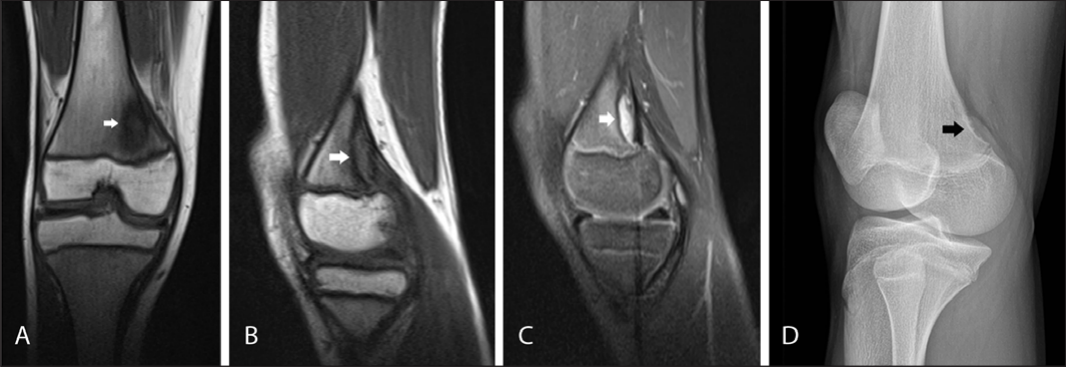
Cortical avulsive irregularity. Coronal **(A)** and sagittal **(B)** T1-WI show a well delineated hypointense defect at the posteromedial femur above the level of the growth plate (arrow). On sagittal FS T2-WI **(C)**, the lesion is of high signal but has a hypointense border (arrow). There is no associated soft tissue mass. Oblique plain film **(D)** shows cortical avulsive irregularity of the knee in another patient. The lesion is typically located at the posteromedial condyle of the femur, above the growth plate of the distal femur. The radiolucent defect is often surrounded by a rim of sclerosis (arrow).

Cortical avulsive irregularity is a benign defect located at the posteromedial condyle of the femur. Previously, it has been designated by the misnomer subperiosteal desmoid, which incorrectly suggests that the lesion is of neoplastic origin. Cortical avulsive irregularity is believed to result from chronic traction either of the medial head of the gastrocnemius muscle or of the insertion of the aponeurosis of the adductor magnus muscle at the posteromedial femoral condyle [3]. It is typically seen in young adolescents around 10 to15–years–old and may be bilateral. Usually, patients do not complain of pain. Physical examination rarely reveals a palpable mass [3]. On plain radiographs, it is located at the distal posteromedial femur above the growth plate and presents as a radiolucent lesion often with some reactive surrounding sclerosis (Figure 3D). On MRI, the lesion is of low signal intensity on T1-weighted images (T1-WI). On T2-WI, the lesion is of high signal intensity and is surrounded by a low signal intensity rim. There may be subtle bone and soft tissue edema (Figure 3A, 3B, 3C). The lesion should not be misinterpreted as an aggressive neoplastic lesion [3].

### Femoral ossification variants mimicking osteochondritis dissecans

**Figure 4:**
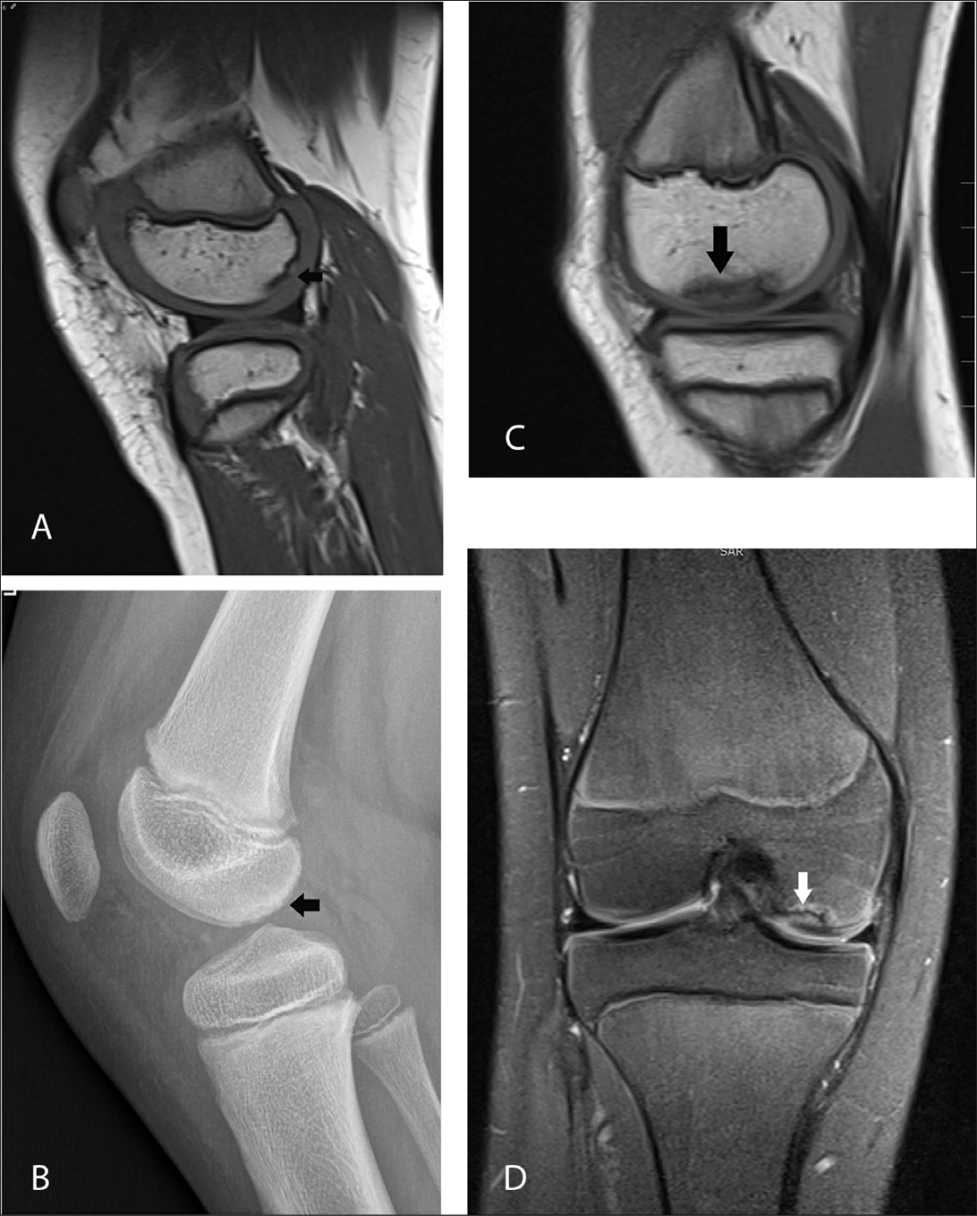
Femoral ossification variant in an 8-year-old boy (A–B) versus osteochondrosis dissecans in a 13-year-old girl (C–D). Sagittal T1-WI **(A)** and plain films **(B)** in the first patient show irregular delineation of the subchondral bone plate of the posterior third of the medial femoral condyle with spiculated borders (arrows). Sagittal T1-WI **(C)** and coronal FS T2-WI **(D)** in the second patient demonstrate a larger lesion which is located in the medial third (black arrow) of the medial femoral condyle. The lesion is surrounded by bone marrow edema (white arrow). The lateral (inner) border of the lesion extends to the intercondylar roof.

Ossification variants of the femoral condyles (Figure 4) are common and should not be confused with osteochondritis dissecans (OCD). They are more frequent in boys than in girls [4]. Useful parameters to distinguish femoral ossification variants from OCD are age (peak age 7–10 years for ossification variants versus 11–14 years for OCD), residual physeal cartilage of more than 30%, location (posterior third of the femoral condyle in ossification variants versus inner third in OCD), absence of intercondylar extension, absence of perilesional bone marrow edema and a lesion angle on coronal images of less than 105 degrees [5].

## Failure to Correlate with Clinical Findings

Some imaging findings are incidental (e.g., age-related degeneration) and do not correlate with the patient’s symptoms. Although a systematic approach of all anatomical knee structures is recommended, imaging findings should always be correlated with the patient’s symptoms in order to avoid overdiagnosis and potentially harmful and unnecessary treatment. It is probably appropriate to describe all MRI abnormalities within the radiological report, but to summarize only those findings which have a high probability of clinical significance in the conclusion of the report. Whenever MRI findings are equivocal, this should be clearly emphasized within the report [6].

Asymptomatic meniscal tears are common, particularly if they are horizontally or obliquely oriented. Borderline or subtle MRI findings that are equivocal for a meniscal tear remain another common problem in daily practice [6,7]. Radial, vertical, complex, or displaced meniscal tears and abnormalities of the collateral ligaments, pericapsular soft tissues, and bone marrow are more seen in symptomatic patients and thus clinically significant [8].

**Figure 5:**
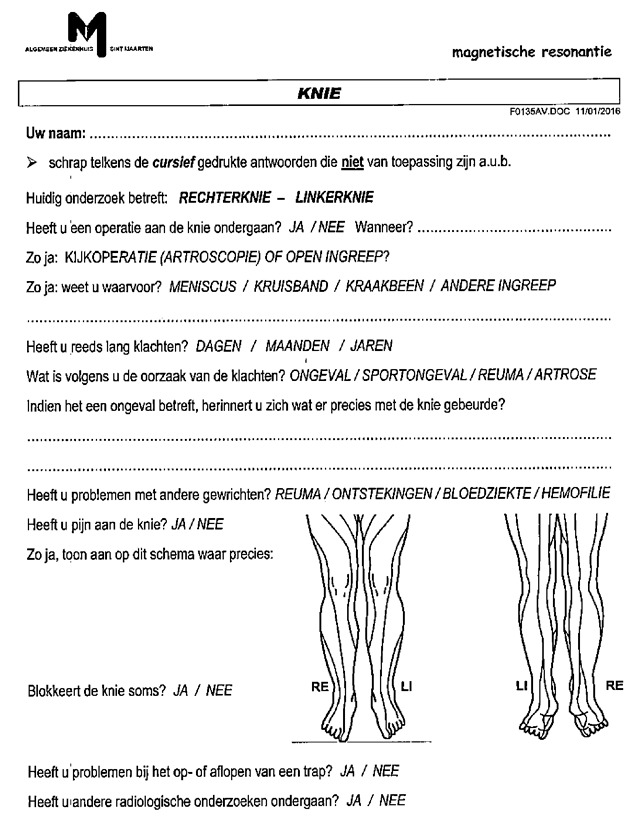
Example of a questionnaire to be filled out by the patient, before the MRI examination. This often provides useful additional information allowing the radiologist to make a confident diagnosis and report on the clinical significant abnormalities.

As request forms for radiological investigations are often incomplete (e.g., not mentioning the precise location of the patient’s pain nor details on previous surgery), we recommend that every patient undergoing an MRI examination should fill out a screening questionnaire indicating previous clinical history (e.g., trauma, previous surgery to cruciate ligaments, menisci, cartilage, rheumatic diseases), location and duration of pain, aggravating activities, previous imaging, etc. (Figure 5).

## Failure to Correlate with Other/Previous Imaging

Most patients that are referred for MRI already underwent previous imaging (plain radiography, ultrasound or MRI). Too often these examinations are disregarded, particularly if they have been performed in an outside institution. We highly recommend looking at previous imaging in the picture archiving and communication system (PACS) or urge the patient to provide the radiologist with examinations done in outside institutions, as comparison with these examinations can be extremely helpful in obtaining the correct diagnosis.

**Figure 6:**
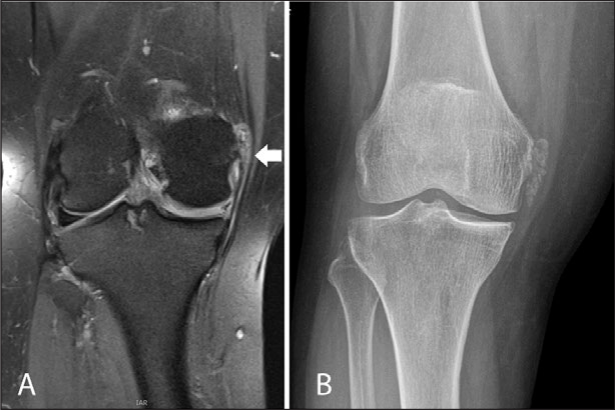
Hydroxyapatite deposition disease (HADD) in the medial collateral ligament in a 66-year-old patient with pain and swelling at the medial aspect of the right knee. Coronal FS T2-WI **(A)** shows thickening and high signal intensity at the femoral insertion of the medial collateral ligament (arrow). Also note shortening of the body of the medial meniscus, in keeping with a previous medial meniscectomy. Plain films **(B)** show calcifications at the medial collateral ligament, allowing precise characterization as HADD with surrounding inflammation as origin of the patient’s pain. Calcifications are far better demonstrated on plain radiographs compared to MRI.

MRI may be less sensitive and specific for identification of calcifications than plain radiographs (Figure 6). In addition, chondrocalcinosis may mimic meniscal tears.

## Technical Artifacts

Interpretation errors due to technical artifacts such as magic angle phenomenon on imaging with low echo time (TE) [9], truncation artefacts on gradient echo imaging [10], blurring artifact on fast spin echo T2-WI [11], phase-artifacts and susceptibility artifacts [12] will not discussed in this short overview.

## Satisfaction of Search

**Figure 7:**
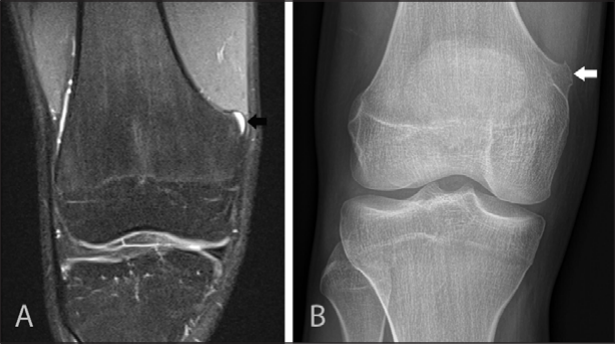
Small cartilaginous exostosis at the medial distal femur of the right knee. Coronal FS T2-WI **(A)** demonstrates a focal contour deformation of the medial distal femur with a small overlying hyperintense cartilage cap (black arrow). Plain film **(B)** confirms a small sessile cartilaginous exostosis (white arrow), which was causing symptoms due to friction with the overlying vastus medialis muscle. This finding was initially overlooked and the patient’s complaints were attributed to meniscal degeneration (which was in retrospect not responsible for the patient’s symptoms). This case also illustrates the usefulness of correlation of MRI with other imaging modalities such as plain films.

Detection of one abnormality may reduce the detectability of another abnormality. Although additional findings are not always clinically significant, some abnormalities may have an impact on the treatment of the patient (Figure 7). A typical example of an undiagnosed finding is a ramp lesion of the medial meniscus, which is often associated with lesions of the anterior cruciate ligament (ACL). A meniscal ramp lesion may either result from disruption of the meniscotibial ligaments of the posterior horn of the medial meniscus, or by disruption of the peripheral attachment of the posterior horn of the medial meniscus [13]. To overcome the phenomenon of satisfaction of search, we recommend a systematic approach in the analysis of all intra- and extra-articular structures of the knee joint.

## Menisci

### Meniscal pseudotears

**Figure 8:**
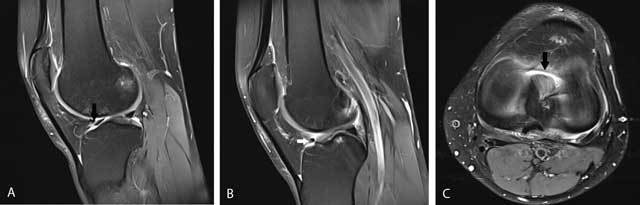
Anterior intermeniscal ligament mimicking a tear of the anterior horn of the lateral meniscus. Fluid (black arrow) between the attachment of the intermeniscal ligament and the anterior horn of the lateral meniscus mimicking a meniscal tear on a sagittal FS T2-WI **(A)**. Analysis of adjacent sagittal slices more medially **(B)** and axial images **(C)** shows that the pseudotear is caused by the course of the anterior intermeniscal ligament (white and black arrows respectively on B and C).

**Figure 9:**
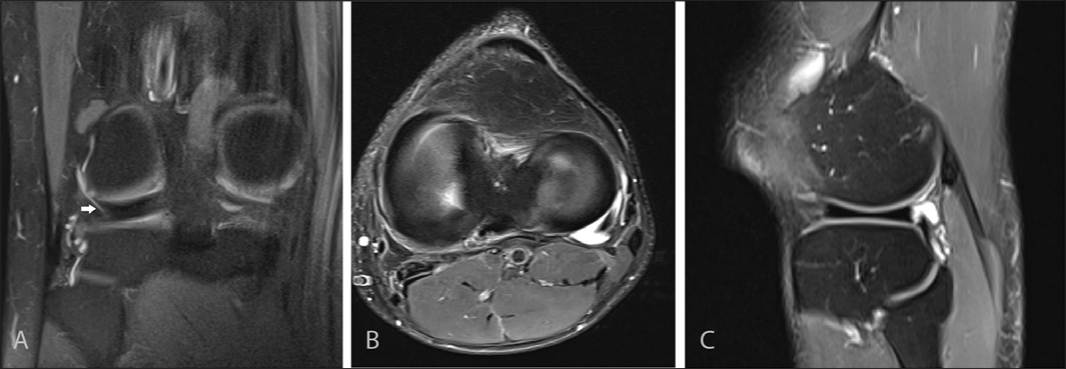
Fluid-filled popliteus recess mimicking a tear of the posterolateral meniscus. Coronal FS T2-WI **(A)** shows a fluid-filled popliteus recess mimicking a peripheral tear of the posterolateral meniscus (white arrow). Analysis of the axial **(B)** and sagittal images **(C)** as well as the typical location allows correct diagnosis of a pseudotear.

Intermeniscal connections (e.g., anterior intermeniscal or geniculate ligament) (Figure 8), ligamentous attachments to the medial and lateral meniscus and partial volume effect of the popliteal recess at the posterolateral meniscus [14] (Figure 9) may be misinterpreted as meniscal tears. The medial posterior femoral recess or the medial gastrocnemius bursa against the posteromedial meniscus are often misinterpreted by the non-experienced reader as meniscocapsular separation. A thorough knowledge of the intra-articular anatomy of the knee is of utmost importance for correct interpretation [15].

### Meniscocapsular separation

**Figure 10:**
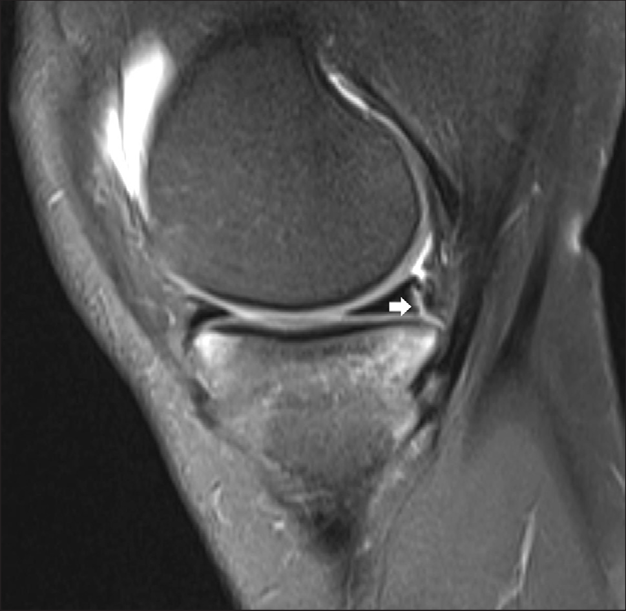
Meniscocapsular separation. Sagittal FS T2-WI shows a fluid-filled irregular delineated cleft at the junction of the posterior horn of the medial meniscus and the adjacent capsule (white arrow). Also note adjacent bone marrow edema within the tibia supporting a posttraumatic origin of the lesion.

The normal fibrovascular peripheral border (also known as the red zone) of the meniscus should not be confused with a peripheral meniscocapsular separation. The red zone is of intermediate signal intensity on T2-WI. In meniscocapsular separation, an abnormal high T2-signal (similar to fluid) is seen between the meniscus and the capsule or within the peripheral zone of the meniscus. It is often accompanied with irregular meniscal borders and meniscal displacement (Figure 10) [15,16].

The precise origin of a so-called meniscal ramp lesion either resulting from disruption of the meniscotibial ligaments of the posterior horn of the medial meniscus or rather by disruption of the peripheral attachment of the posterior horn of the medial meniscus, is still debated [13].

### Meniscal root tears

**Figure 11:**
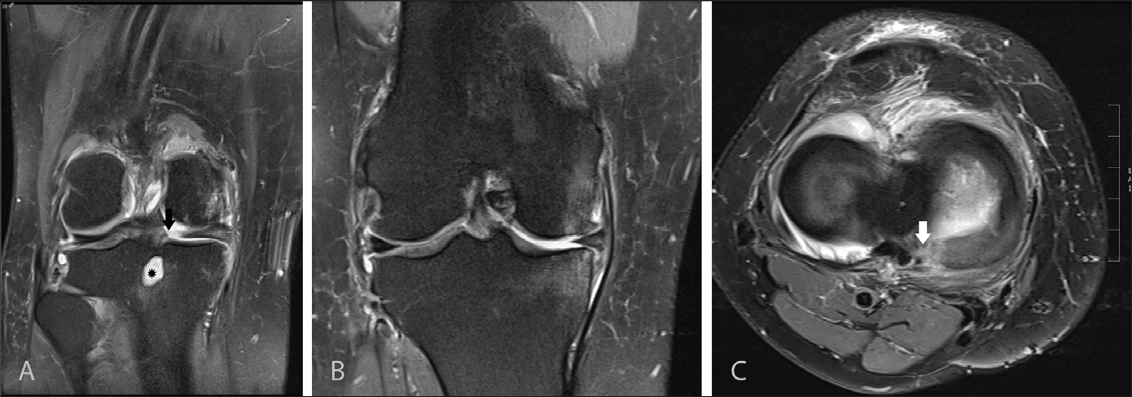
Tear of the posterior medial root. Coronal FS T2-WI at the posterior horn of the medial meniscus **(A)** shows disruption of the posterior medial root (black arrow) and an associated intra-osseous ganglion cyst at the intercondylar part of the posteromedial tibia (black asterisk). Coronal FS T2-WI at the body of the medial meniscus **(B)** shows extrusion of the medial meniscus underneath the medial collateral ligament, adjacent bone marrow edema, cartilage loss and premature osteophyte formation. Axial FS T2-WI **(C)** confirms the radial tear at the posterior medial root (white arrow).

**Figure 12:**
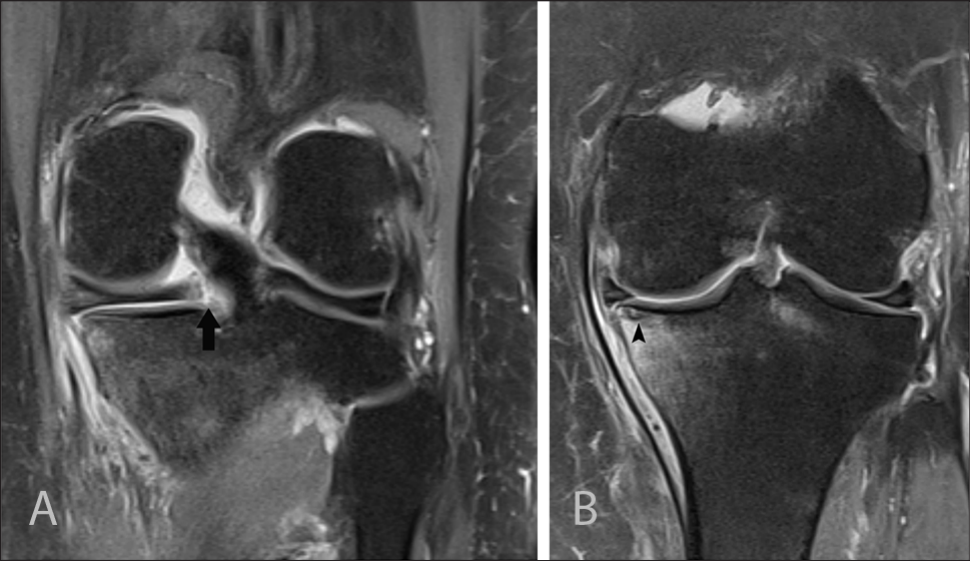
Tear of the posterior medial root and associated subchondral insufficiency fracture. Coronal FS T2-WI at the posterior horn of the medial meniscus **(A)** shows disruption of the posterior medial root (black arrow). Coronal FS T2-WI at the body of the medial meniscus **(B)** shows extrusion of the medial meniscus underneath the medial collateral ligament and a subchondral hypointense band in keeping with a subchondral insufficiency fracture (black arrowhead) with surrounding bone marrow edema.

Meniscal root tears are less common than other types of meniscal tears. Nevertheless, they have an important clinical impact. They are often associated with extrusion of the meniscus with respect to the tibial margin. This may alter the biomechanical forces on the medial femorotibial compartment and predisposes to meniscal extrusion, premature cartilage lesion (Figure 11) and subchondral insufficiency fractures (Figure 12) [15]. Posterior medial root tears are most common compared to other root tears [17].

### Radial tears

**Figure 13:**
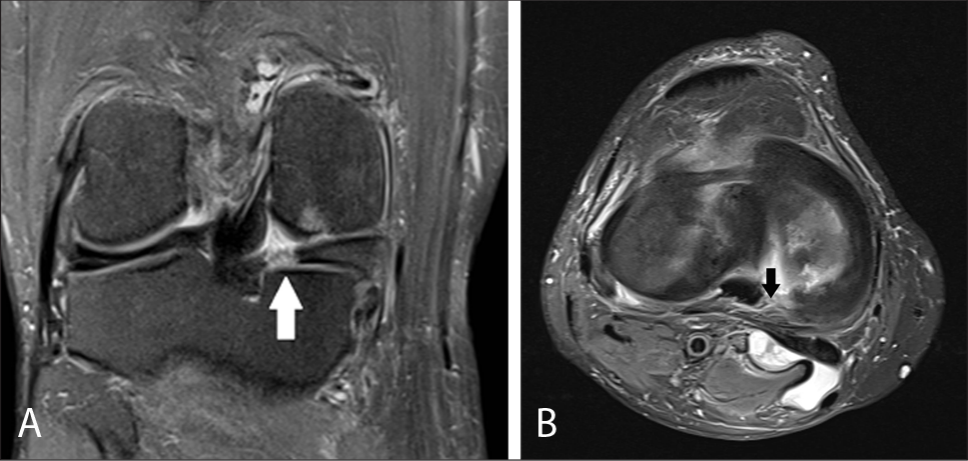
Radial tear of the posterior horn of the medial meniscus. Coronal FS T2-WI at the posterior horn of the medial meniscus **(A)** shows a tear of the inner third of the posterior horn of the medial meniscus medial to the posterior medial root (white arrow). Axial T2-WI at the body of the medial meniscus **(B)** shows irregular delineation of the tear (black arrow). Part of the inner segment of the medial meniscus is missing.

Radial tears result from a high impact and have a perpendicular course to the long axis of the meniscus. They occur initially at the free edges of the meniscus and may progress through the meniscus, resulting in splitting of the meniscus into separate parts [15]. Radial tears are often subtle and a meticulous analysis of coronal, sagittal and axial images is required to make a confident diagnosis. MRI signs of radial tears are the missing triangle sign, disrupted bow tie or focal absence of the meniscus without displacement of a meniscus fragment (Figure 13).

### Posterior horn tears of the lateral meniscus

**Figure 14:**
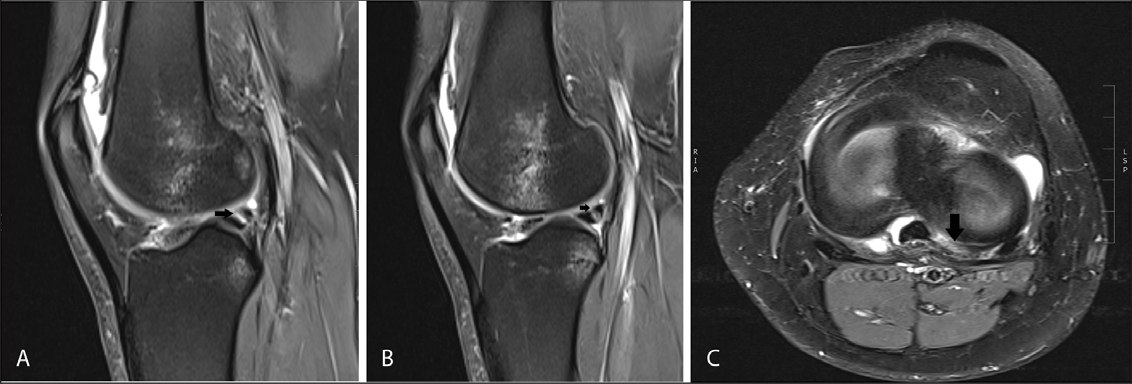
Tear of the posterior horn of the lateral meniscus. Sagittal FS T2-WI at the posterior horn of the lateral meniscus on adjacent slices **(A and B)** shows a fluid-filled tear at the insertion of the posterior meniscofemoral ligament at the inner third of the posterior horn of the lateral meniscus (black arrow). Note also bone marrow edema in the middle third of the lateral femoral condyle and posterolateral tibia due to an ACL tear (not shown on these images). Axial FS T2-WI **(C)** shows an irregularly delineated tear at the periphery of the inner third of the posterior horn of the lateral meniscus with presence of the so-called zip sign (black arrow).

Tears of the posterior horn of the lateral meniscus are difficult to recognize and often underreported. Careful analysis of thin axial images associated with sagittal MR sections is required to detect this often subtle lesion. The *zip* sign on axial images is a useful imaging sign encountered in tears of the posterolateral meniscus, extending from the distal insertion of meniscofemoral ligaments (MFLs) to the posterior horn of the lateral meniscus (Figure 14) [18].

### Displaced meniscal fragments

Although complex tears with displaced meniscal fragments are readily identified, the precise displacement of free meniscal fragments is often underreported or disregarded.

**Figure 15:**
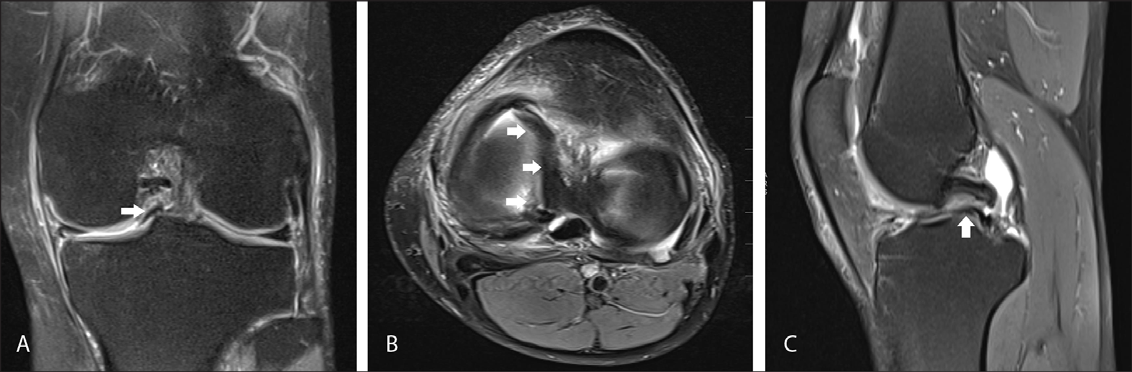
Bucket-handle tear of the medial meniscus. Coronal FS T2-WI **(A)** shows displacement of meniscus fragment to the intercondylar notch (fragment in notch sign) (white arrow). Axial FS T2-WI **(B)** confirms intercondylar displacement of the inner fragment of the medial meniscus (white arrows). Sagittal FS T2-WI **(C)** shows a meniscus fragment underneath the posterior cruciate ligament (double PCL sign, white arrow).

A bucket-handle tear consists of a displaced longitudinal tear (Figure 15). MRI signs on sagittal images include the double posterior cruciate ligament (PCL) sign [19] and absent bow tie sign [20,21]. MRI signs on coronal images include the fragment-in-notch sign [22], the flipped meniscus sign [23] or a centrally located meniscus fragment.

**Figure 16:**
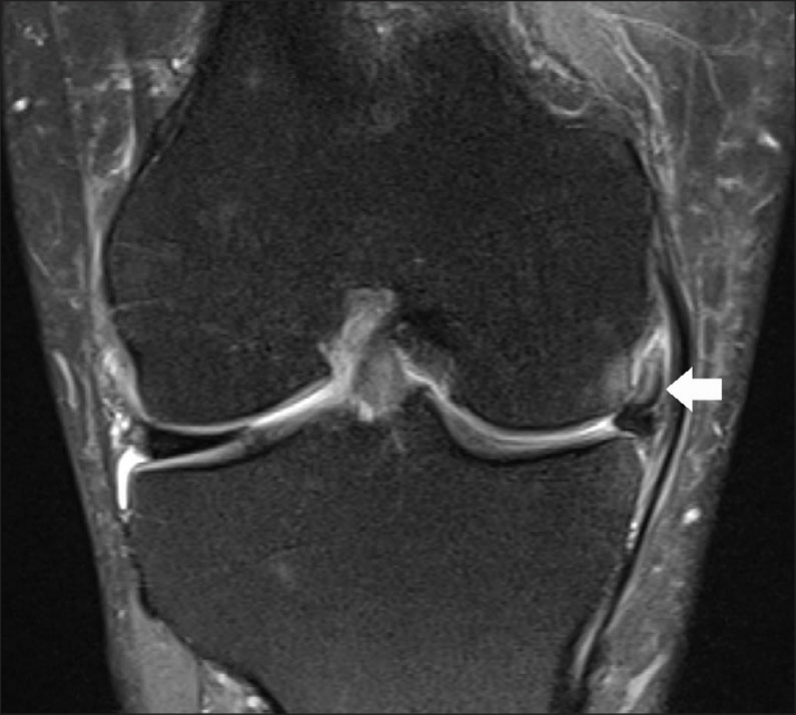
Displaced medial meniscus fragment in the medial meniscofemoral recess. Coronal FS T2-WI demonstrates shortening of the body of the medial meniscus and displacement of a meniscal fragment within the medial meniscofemoral recess (white arrow). Note adjacent bone marrow edema at the medial femoral condyle possibly caused by menisco-osseous impingement.

Fragment displacement underneath the collateral capsuloligamentary structures into the meniscal recess is less frequent [24,25], but these fragments are difficult to identify arthroscopically. Therefore, a correct description provides crucial information for the surgeon [26]. Fragment displacement within the inferior meniscocapsular recess may be accompanied by focal bone marrow edema at the proximal tibia, probably due to menisco-osseous impingement (Figure 16).

### Discoid meniscus

A discoid meniscus is a rather rare variant in the shape of the meniscus, usually involving the lateral meniscus and more rarely the medial meniscus [27]. The patient may be asymptomatic or a snapping sound may be present. Discoid meniscus may also predispose to tears of the involved meniscus, causing pain and swelling.

According to the Watanabe classification, discoid meniscus is divided in three types: type I complete; type II, incomplete; and finally type III or Wrisberg-ligament variant, in which the posterior meniscofemoral attachment is absent resulting in an unstable meniscus with hypermobility [27].

As a rule of thumb, a discoid meniscus should be suspected if three or more contiguous body segments are present or if the size between the free margin and the periphery of the body on coronal images exceeds more than 1.5 cm. More accurate criteria are a ratio of the minimal meniscal width to maximal tibial width (on coronal images) of more than 20% and a ratio of the sum of the width of both lateral horns to the meniscal diameter (on a sagittal slice showing the maximal meniscal diameter) of more than 75% [28].

### Postoperative meniscus

The evaluation of recurrent tears after surgery is much more complex than in the native meniscus because the imaging criteria for evaluation of meniscal tears in the native meniscus (abnormal signal on two MR slices and abnormal meniscal shape) are not reliable at the location of the meniscectomy. Detailed correlation with the type of surgery and preoperative imaging is mandatory. Discussion of the postoperative meniscus is beyond the scope of this short review and we refer to a recent review on the topic [29].

## Cruciate Ligaments

### Partial versus complete anterior cruciate ligament tears

MRI evaluation of a partial anterior cruciate ligament (ACL) tear and differentiation from a complete ACL tear, mucoid degeneration or even a normal ACL can be challenging because of overlapping imaging features [30].

Partial tears of the femoral origin of the ACL may be particularly challenging on sagittal images alone. Therefore, meticulous correlation of axial and coronal images for assessment of the degree of ligament fiber disruption is mandatory [31].

MRI has an overall moderate accuracy to distinguish stable from unstable ACL tears. ACL discontinuity and abnormal orientation of ACL fibers have an accuracy of 79% and 87% respectively. Although anterior tibial translation, uncovering of the posterior horn of the lateral meniscus, and hyperbuckled PCL are specific signs of an unstable tear, the sensitivity of these signs is as low as 23% [32]. Bone marrow edema around the lateral knee compartment is not a good parameter for predicting stability [32].

### Ganglion cyst and mucoid degeneration of the anterior cruciate ligament

**Figure 17:**
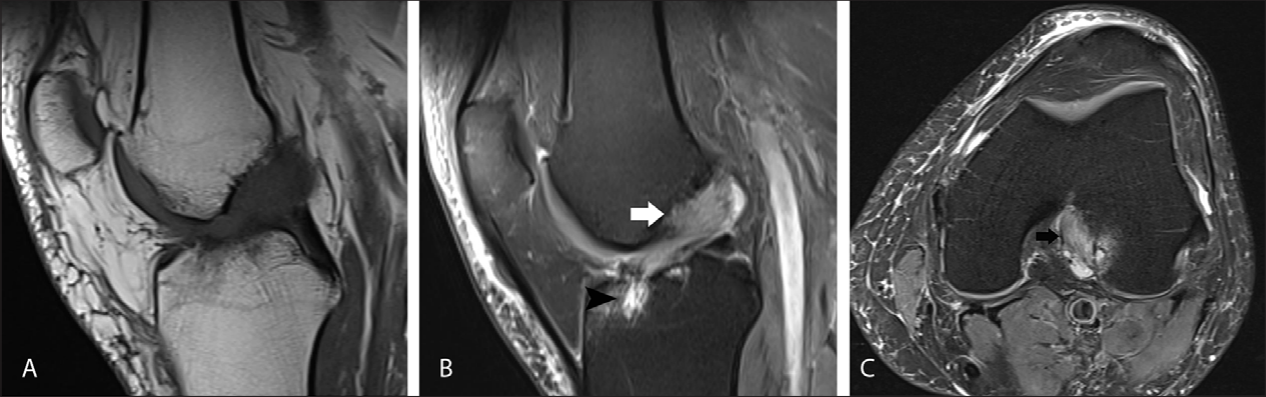
Mucoid degeneration of the anterior cruciate ligament (ACL). On sagittal T1-WI **(A)**, the ACL is of intermediate signal intensity and the ligamentous structure has disappeared. On sagittal FS T2-WI **(B)**, the ACL has a striated pattern with interspersed intact ligamentous fibers, resembling a celery stalk (white arrow). There is also an intraosseous ganglion cyst at the tibial insertion of the ACL (black arrowhead). Axial FS T2-WI **(C)** demonstrates the intermediate signal of the ACL with interspersed intact ligamentous fibers (black arrow).

Thickening and increased T2-signal may also be seen in ganglion cysts or mucoid degeneration of cruciate ligaments, but the pattern is often more striated on T2-WI with interspersed intact ligamentous fibers, resembling a celery stalk. There is often associated bone marrow edema and/or intraosseous ganglion cyst formation at the femoral and tibial insertion of the ACL [33]. On T1-WI, the ACL is of intermediate signal and the ligamentous structure has disappeared (Figure 17). Typically, there is no history of previous trauma.

## Analysis of Abnormal Bone Marrow Pattern and Determination of its Etiology

**Figure 18:**
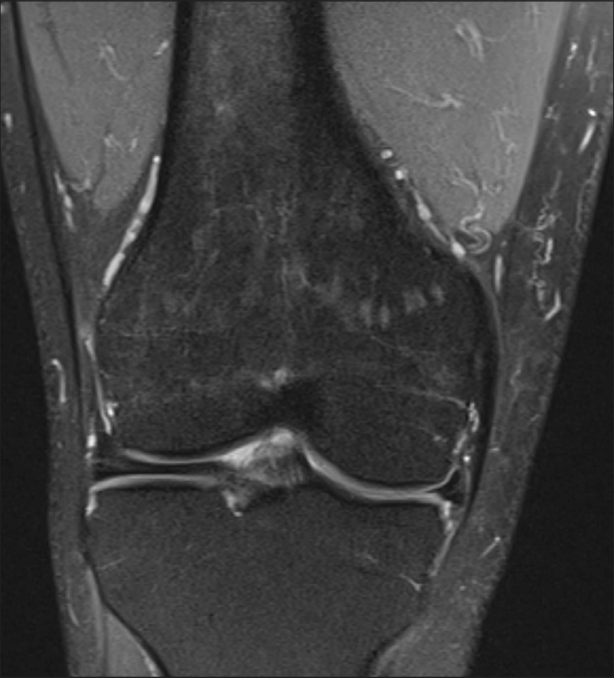
Residual red bone marrow. Coronal FS T2-WI shows multiple small island of residual red bone marrow of intermediate signal in the distal femur.

**Figure 19:**
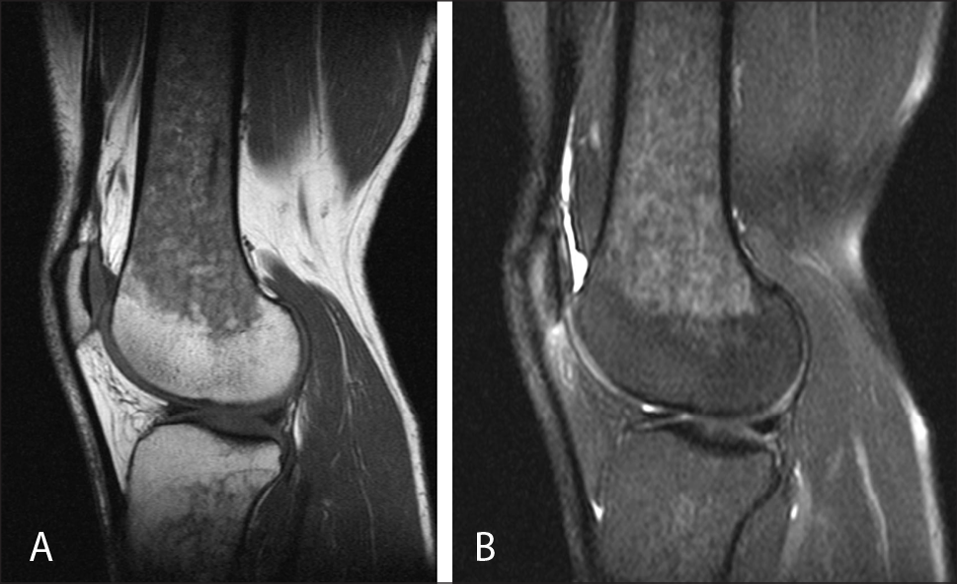
Bone marrow reconversion. Sagittal T1-WI **(A)** and FS T2-WI **(B)** shows bone marrow of intermediate signal intensity in the distal femoral diaphysis and proximal tibia in a middle-aged heavy smoking female patient.

Small residual islands of red bone marrow are often correctly characterized as physiological by most radiologists (Figure 18), but larger focal areas of physiological bone marrow reconversion may be more worrisome (Figure 19). Correlation with age, female gender, heavy smoking, sports activities with increases oxygen debt (e.g. distance running, free diving), obesity, diabetes, other diseases causing chronic anemia and finally treatment with hematopoietic growth factors is mandatory [34].

Abnormal bone marrow about the knee has a variable etiology including acute or chronic trauma [35], degenerative joint disease [36] and tumoral causes. Although bone marrow edema (BME) is readily identified, less experienced radiologists often struggle with precise identification of the underlying etiology. BME due to avulsion injuries is often more subtle than bone marrow edema due to compression injuries [35]. Identification of the BME pattern and location helps to identify the mechanism of trauma [37,38].

**Figure 20:**
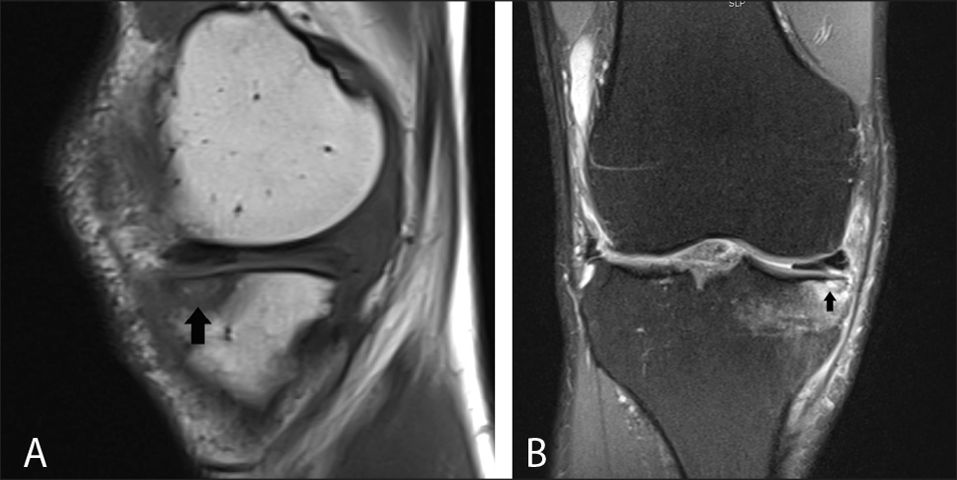
Subchondral stress fracture initially misinterpreted as an osteochondral fracture. Sagittal T1-WI **(A)** and coronal FS T2-WI **(B)** showing a subchondral hypointense band-like structure (arrow) with surrounding bone marrow edema on FS T2-WI.

**Figure 21:**
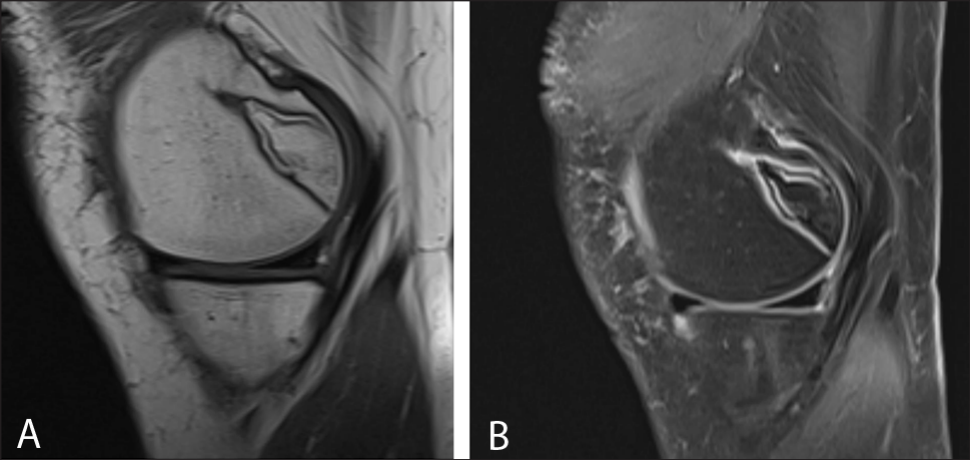
Typical bone infarct. Sagittal T1-WI **(A)** and sagittal FS T2-WI **(B)** showing a well-demarcated area in the posteromedial condyle of the femur.

Subchondral insufficiency fractures are often not correctly reported by the less experienced radiologist and may be misinterpreted as osteochondral fractures (Figure 20) or avascular necrosis (Figure 21). These lesions result often from altered biomechanics and may be seen in patients with recent meniscectomy on the involved site [39].

## Correct Grading of Cartilage Lesions

Correct grading of cartilage lesions on conventional MRI using the arthroscopic classification grading system remains a difficult task and there is much interobserver variability.

**Figure 22:**
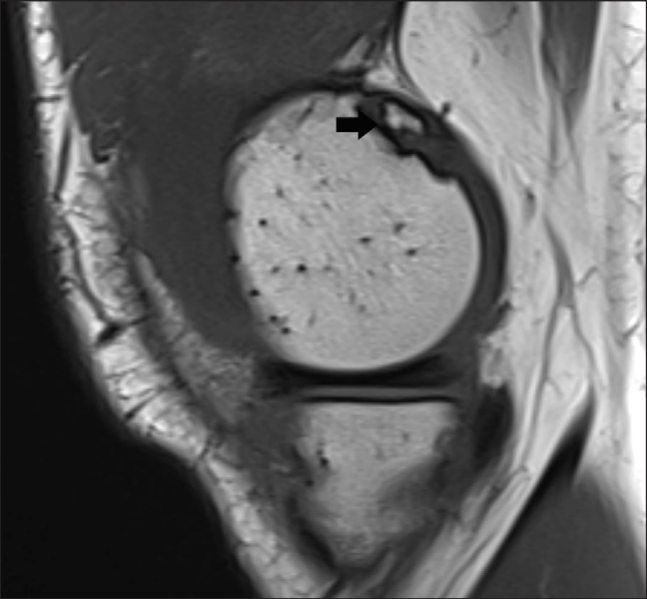
Loose bodies. Sagittal T1-WI shows small loose body adjacent to the posterior medial femoral condyle (black arrow).

Identification of loose bodies is often disregarded, particularly if they are adjacent to bone of ligamentous structures (Figure 22).

## Cystic Lesions about the Knee

There is a variety of cystic lesions and cyst mimickers about the knee of which the terminology is often confused and inappropriately used.

### Joint recesses

**Figure 23:**
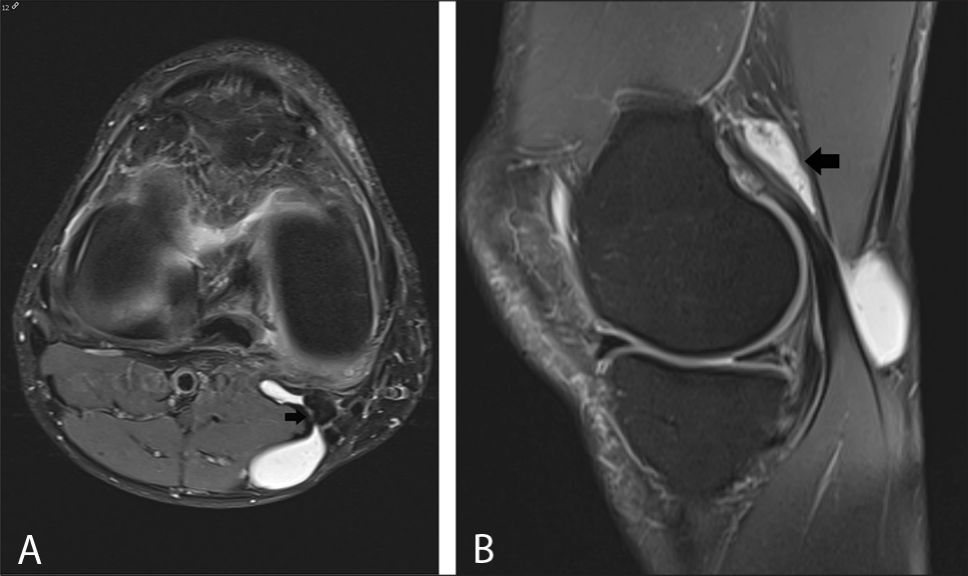
Enlarged medial gastrocnemius-semimembranosus recess (Baker’s cyst). On axial FS T2-WI **(A)**, a Baker cyst is located posteromedially and has a connecting stalk (black arrow) with the knee joint between the medial gastrocnemius and semimembranosus tendon. On sagittal FS T2-WI **(B)**, a Baker cyst has often a considerable extension above the joint space (black arrow).

Joint recesses are normal outpouchings of the joint cavity, which may enlarge in case of a joint effusion. The prototype of a joint recess is the medial gastrocnemius-semimembranosus recess, which is located posteromedially in the knee. It has a typical connecting stalk with the knee joint between the medial gastrocnemius muscle and the semimembranosus tendon. Expansion gives rise to a so-called Baker’s cyst (Figure 23).

**Figure 24:**
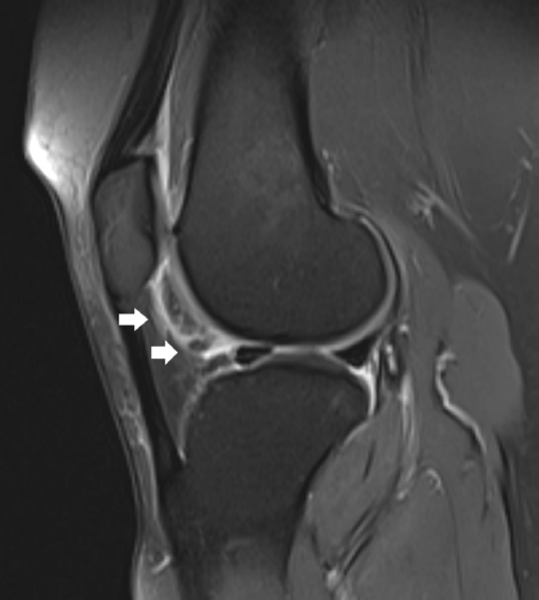
Ligamentum mucosum. Sagittal FS T2-WI showing fluid-filled signal along the course of the infrapatellar plica (white arrows).

The popliteus recess is located posterolaterally and may be the source of a pseudotear of the posterolateral meniscus due to volume averaging (Figure 9). Distention of the ligamentum mucosum (infrapatellar plica) anteriorly within Hoffa’s fad pad is rarer (Figure 24). The significance of this finding is not clear. It may result from chronic stress or represent a variant of Hoffa’s disease [40].

### Bursae

**Figure 25:**
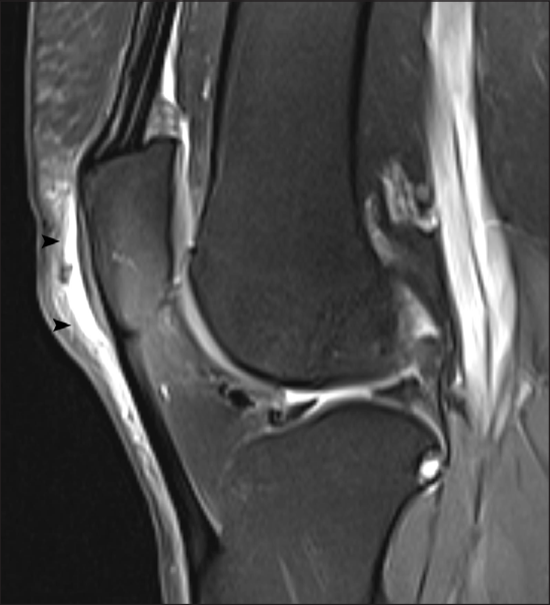
Distention of the prepatellar bursa. Sagittal FS T2-WI showing thickening and increased fluid within the prepatellar bursa (black arrowheads).

**Figure 26:**
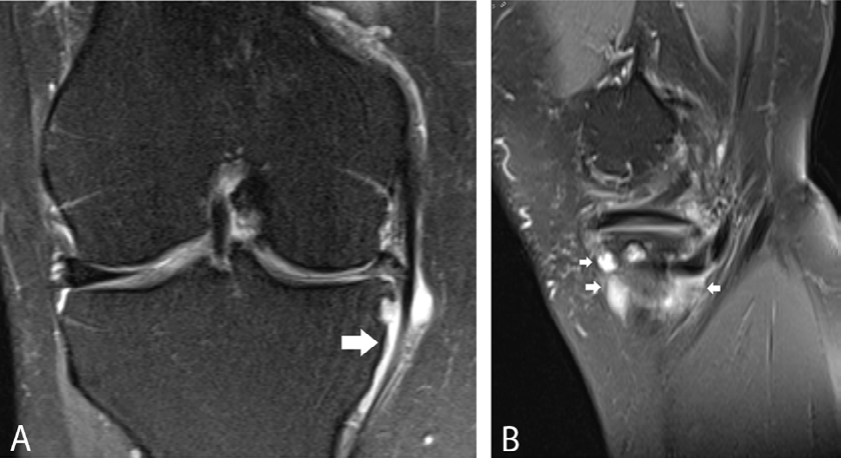
Pes anserinus bursa. Coronal FS T2-WI **(A)** and sagittal FS T2-WI **(B)** showing a multiloculated fluid-filled structure adjacent to the pes anserinus tendon. The lesion is typically located underneath the joint space (white arrows).

**Figure 27:**
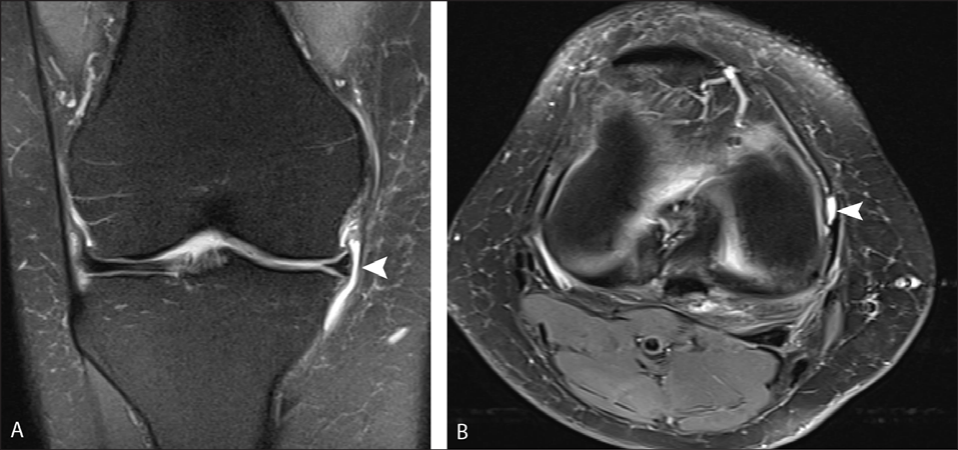
Medial collateral ligament bursa. Coronal FS T2-WI **(A)** and axial FS T2-WI **(B)** showing a well-delineated fluid-filled structure between the deep and superficial layers of the medial collateral ligament (white arrowheads).

Bursae are synovial-lined structures that decrease friction between moving structures. They may become distended due to (repetitive) trauma, inflammatory or infectious disease (e.g., rheumatoid arthritis, crystal deposition disease) or tumor or tumor-like conditions (e.g., pigmented villonodular synovitis, chondromatosis). Around the knee, bursae are characterized by their specific location, shape and relationship with surrounding structures. The following bursae are often found around the knee: prepatellar bursa (Figure 25), superficial infrapatellar bursa, deep infrapatellar bursa, pes anserinus bursa (Figure 26), medial collateral ligament bursa (Figure 27), semimembranosus – tibial collateral ligament bursa. A fibular collateral ligament – biceps femoris bursa is much rarer.

### Synovial cysts and ganglion cysts

**Figure 28:**
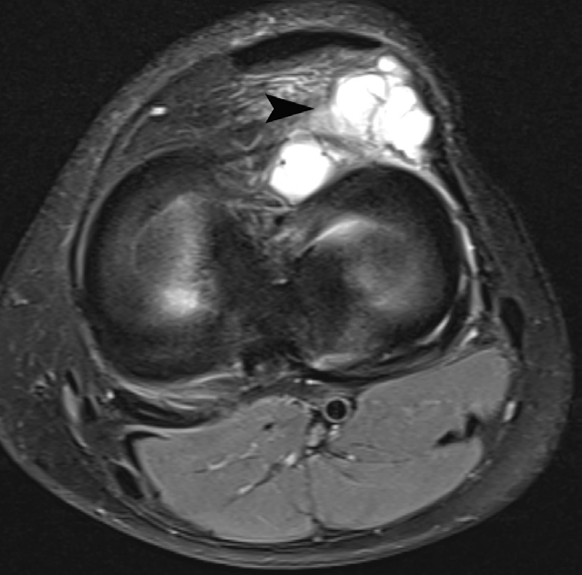
Ganglion cyst adjacent to the anterior horn of the lateral meniscus. Axial FS T2-WI showing a multiloculated cyst adjacent to the anterior horn of the lateral meniscus (black arrowhead).

Ganglion cysts (Figure 28) may be located anywhere around the joints. They may either have a communicating stalk with the knee joint or rather be remote from the joint without any visible communication. Special forms of ganglion cysts include meniscal cysts, cruciate ligament cysts, intraosseous ganglia, cystic adventitial disease and peri- or intraneural cysts.

**Figure 29:**
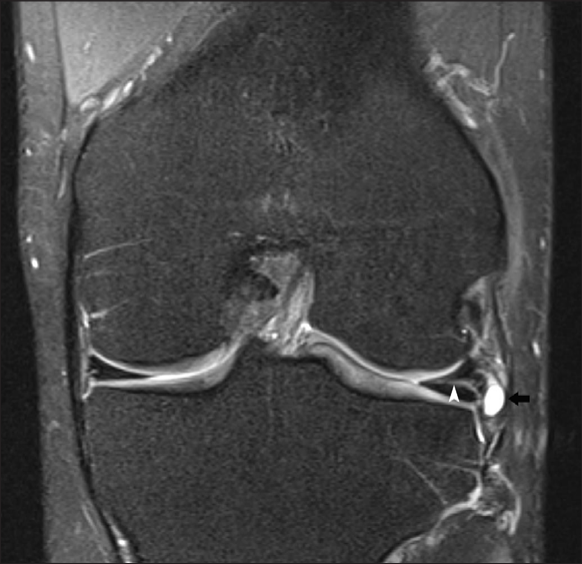
Lateral meniscus cyst. Coronal FS T2-WI showing a horizontal lateral meniscus tear (white arrowhead) with extension to a small lateral meniscal cyst (black arrow).

Meniscal cysts consist of a collection of synovial fluid, which is extruded through a meniscal tear. Lateral meniscal cysts are usually located at the periphery of the middle third of the meniscus, whereas medial meniscal cysts may present at a distant location from the joint because of the firm attachment of the medial meniscus to the joint capsule. The identification of an associated meniscal tear and communication of the cyst with the tear is the key to the characterization of a meniscal cyst (Figure 29).

Cruciate ligament cysts occur within the fibers or on the surface of the cruciate ligaments (ACL – PCL). They have a similar appearance as mucoid degeneration of the cruciate ligaments (Figure 17).

**Figure 30:**
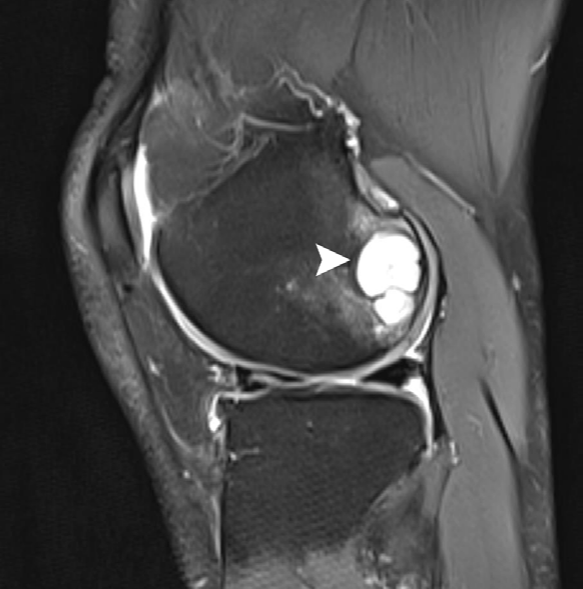
Intraosseous ganglion cyst. Sagittal FS T2-WI showing a well-delineated fluid-filled lesion at the posterolateral femoral condyle (white arrowhead). There is some adjacent bone marrow edema.

Intraosseous ganglia (Figure 30) are intraosseous extensions from synovial fluid through the subchondral bone.

Cystic adventitial disease is a ganglion cyst which is located in the wall of vessel (e.g., popliteal artery).

### Cyst mimickers

Not all masses which display very high signal intensity on T2-WI are necessarily fluid-filled. Tumors mimicking cysts include both benign (e.g., peripheral nerve sheath tumors, myxomas) and malignant tumors with prominent areas of necrosis or myxoid degeneration (e.g., myxoid liposarcoma).

Intravenous contrast should be administered whenever there is doubt about the cystic or solid nature of the visualized mass. Furthermore, abscesses and vascular masses, such as varices and popliteal artery aneurysms may simulate cystic lesions. For a more detailed discussion of cystic lesion about the knee, we refer to specific articles on this subject [41,42].

## Conclusion

Although potential pitfalls are numerous in the interpretation of MRI of the knee, a thorough knowledge of anatomy and its variations, close correlation with age, previous medical history and symptoms and systematic imaging approach will avoid interpretation errors. Specific recommendations are summarized in the Table 1.

**Table 1:** Recommendations to avoid common mistakes in assessment of MRI of the knee.


Consider age and clinical findings and ask for any previous history before interpreting the images. Make use of standardized questionnaires to indicate the location of the pain, duration of the complaints, history of trauma, aggravating activities, underlying diseases, previous surgery, etc. Look at previous imaging studies and other imaging modalities (such as plain films) Be aware of normal variants and developmental anatomy Scrutinize the menisci for common pitfalls Analyze all images systematically for all structures and all pulse sequences (T1-WI and T2-WI; sagittal, axial and coronal images). Bone marrow edema is a nonspecific finding with a variable etiology that may be of traumatic, degenerative or tumoral origin. Always look for other abnormalities (e.g. pigmented villonodular synovitis, gout, other crystal deposition diseases, bone marrow abnormalities) than common intra-and peri-articular pathology (e.g. menisci, anterior cruciate ligament, cartilage)

